# New hopes and promises in the treatment of ovarian cancer focusing on targeted treatment—a narrative review

**DOI:** 10.3389/fphar.2024.1416555

**Published:** 2024-06-14

**Authors:** Małgorzata Satora, Krzysztof Kułak, Bartłomiej Zaremba, Arkadiusz Grunwald, Paulina Świechowska-Starek, Rafał Tarkowski

**Affiliations:** ^1^ 1st Chair and Department of Oncological Gynecology and Gynecology, Students’ Scientific Association, Medical University of Lublin, Lublin, Poland; ^2^ 1st Chair and Department of Oncological Gynaecology and Gynaecology, Medical University of Lublin, Lublin, Poland; ^3^ Primary Care Clinic, Non-Public Healthcare Facility Medycyna 2001, Lublin, Poland

**Keywords:** ovarian cancer, targeted treatment, angiogenesis inhibitors, folate receptor inhibitors, PARP inhibitors, bevacizumab, immune checkpoint inhibitors immunotherapy

## Abstract

Unfortunately, ovarian cancer is still diagnosed most often only in an advanced stage and is also the most lethal gynecological cancer. Another problem is the fact that treated patients have a high risk of disease recurrence. Moreover, ovarian cancer is very diverse in terms of molecular, histological features and mutations. Many patients may also develop platinum resistance, resulting in poor response to subsequent lines of treatment. To improve the prognosis of patients with ovarian cancer, it is expected to make better existing and implement new, promising treatment methods. Targeted therapies seem very promising. Currently, bevacizumab - a VEGF inhibitor and therapy with olaparib - a polyADP-ribose polymerase inhibitor are approved. Other methods worth considering in the future include: folate receptor α, immune checkpoints or other immunotherapy methods. To improve the treatment of ovarian cancer, it is also important to ameliorate the determination of molecular features to describe and understand which group of patients will benefit most from a given treatment method. This is important because a larger group of patients treated for ovarian cancer can have a greater chance of surviving longer without recurrence.

## 1 Introduction

Despite continuous progress in gynecological oncology, statistics are still unfavorable for ovarian cancer. It is the third most common gynecological cancer in the world with the highest mortality rate among cancers of the female reproductive system (Höhn et al., 2020). Moreover, according to Global Cancer Statistics 2020, up to 24,000 women will be diagnosed with ovarian cancer every year (Sung et al., 2021). Finally, most of these patients learn about the disease only in its advanced stage, where the 5-year survival rate is less than 30% (Zachou et al., 2023).

The goal of primary ovarian cancer treatment is surgical removal of the tumor and assessment of the cancer’s advancement along with possible adjuvant chemotherapy. The emphasis is placed not only on prolonging survival and delaying relapse, but also on improving the woman’s quality of life, which also has a significant impact on the effectiveness of treatment. Unfortunately, it turns out that up to 70% of patients treated with standard platinum chemotherapy will have a recurrence of the disease within 18–28 months (Armstrong et al., 2021; Fan et al., 2023).

**FIGURE 1:**
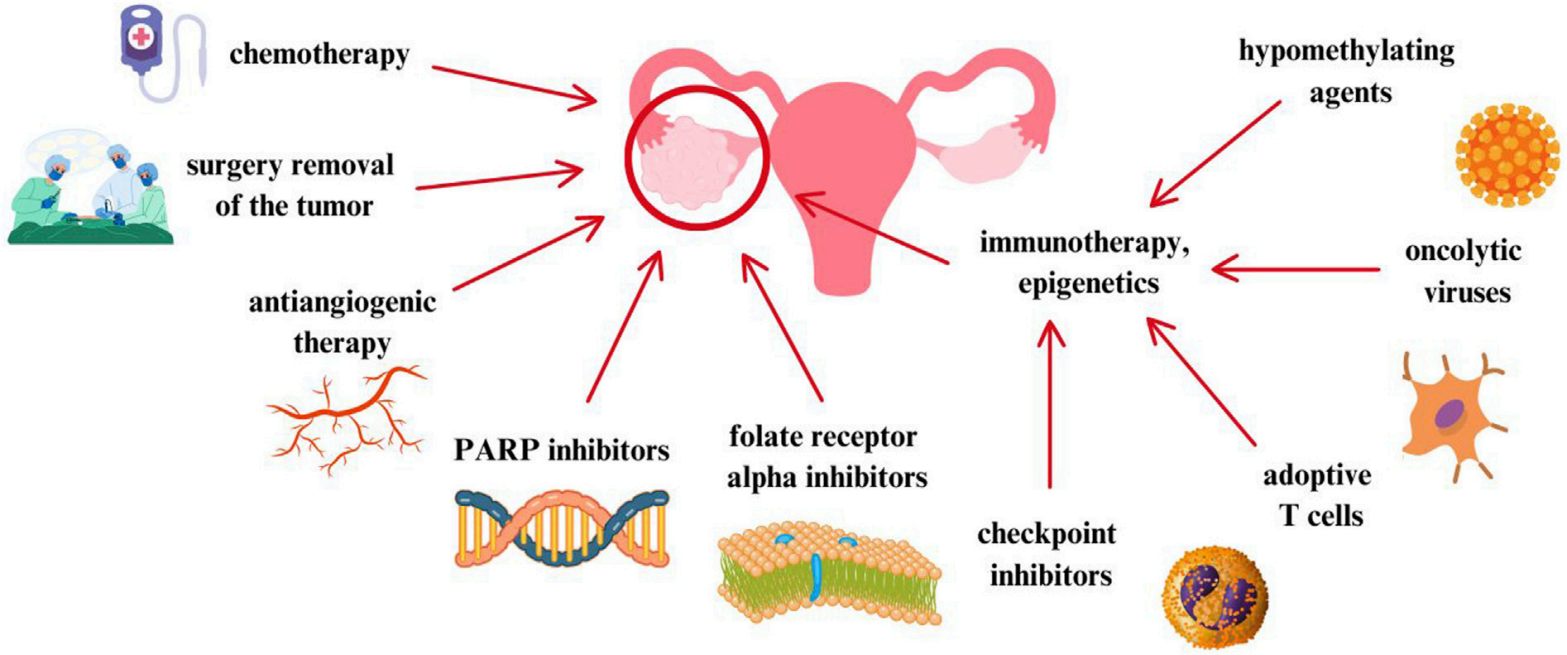
Ovarian cancer treatment methods described in the article (Burger et al., 2011; Coleman et al., 2017a; Slade, 2020; Arend et al., 2021; Heo, 2023).

Considering these alarming data, it is extremely important to develop new treatment methods and conduct further randomized clinical trials. New therapies and treatment strategies are based on molecular features, tumor cell proliferation, escape from immune surveillance or death signals. For this purpose, increasingly well-known standards have become the subject of discussion and interest, such as: antiangiogenic therapy with bevacizumab. The key here is to inhibit VEGF and thus the proliferation of endothelial cells (Coleman et al., 2017a). 15% of women with ovarian cancer have a BRCA1 and/or BRCA2 mutation (Slade, 2020). In these patients, poly(ADP-ribose) polymerase (PARP) inhibitors are used, which are a promising treatment method for women with this mutation. Although both angiogenesis inhibitors and PARP inhibitors have benefits, unfortunately they only delay the recurrence of ovarian cancer. Moreover, it turns out that immune checkpoint inhibitors are also not associated with benefits for patients with ovarian cancer (Arend et al., 2021). In turn, folate receptor alpha (FRα) is expressed in tissues on the plasma membrane of epithelial cells of the ovary and fallopian tube. Mirvetuximab soravtansine, a folate receptor inhibitor, is approved by the FDA for the treatment of women with platinum-resistant ovarian cancer (Heo, 2023). In Figure 1, ovarian cancer treatment methods described in the article are performed.

Although none of the methods described above has yet cured ovarian cancer, it is important to develop more and more clinical trials to improve the therapy and quality of life of patients with ovarian cancer. In this narrative review, we discuss and evaluate the latest treatments for ovarian cancer. We made a detailed review of angiogenesis inhibitors, folate receptor inhibitors, PARP inhibitors and, finally, immunotherapy. We believe that the following work will provide valuable tips for gynecologists and oncologists in selecting the best treatment strategy for patients.

## 2 Methods

A search was performed in January 2024 with no time restrictions for searching articles. The studies cited in this review were selected from the PubMed, Scopus and Google Scholar databases. Terms used by us to find articles were created by combining all words connected with ovarian cancer and available treatment methods by using Boolean operator “OR”. We used keywords: ovarian cancer, bevacizumab, cediranib, nintedanib, pazopanib, olaparib, niraparib, rucaparib, mirvetuximab soravtansine, farletuzumab, vintafolide, checkpoint inhibitors, adoptive T cell transfer, therapeutic vaccines, oncolytic viruses. Moreover, we also used more specific terms relating to epidemiology and etiology of ovarian cancer, using “epidemiology” and “etiology”.

Our aim was to create a narrative review, however, we used a paper selection to find appropriate articles. The inclusion criteria were studies evaluating the treatment of ovarian cancer, manuscripts written in English, retrospective studies, clinical trials and metanalyses. The exclusion criteria were manuscripts that did not investigate the treatment of ovarian cancer, articles not written in English, conference abstracts, document types including review and systematic review, technical report, editorial, letter and duplicated papers. Manuscripts with non-available full-text were also not taken into account.

## 3 Antiangiogenic therapy

Malignant tumors are characterized by uninhibited cell proliferation, which leads to the formation and spread of metastases. In this tumor development, cancer cells require the supply of oxygen and nutrients, which leads to the induction of angiogenesis. This process is the creation of new blood vessels from existing ones, thanks to which the metabolic needs of the tumor are met (Carmeliet and Jain, 2000; Ferrara et al., 2004; Burger et al., 2011; Xu et al., 2012; Li et al., 2016; Hegde et al., 2018). Angiogenesis promotes tumor progression and worse prognosis, including ovarian cancer. Therefore, antiangiogenic therapy has been the subject of interest in numerous clinical trials for over 20 years (Burger et al., 2011). In this chapter, we analyzed the latest and most important research on the use and effectiveness of antiangiogenic therapy in the treatment of ovarian cancer.

### 3.1 Bevacizumab

**FIGURE 2:**
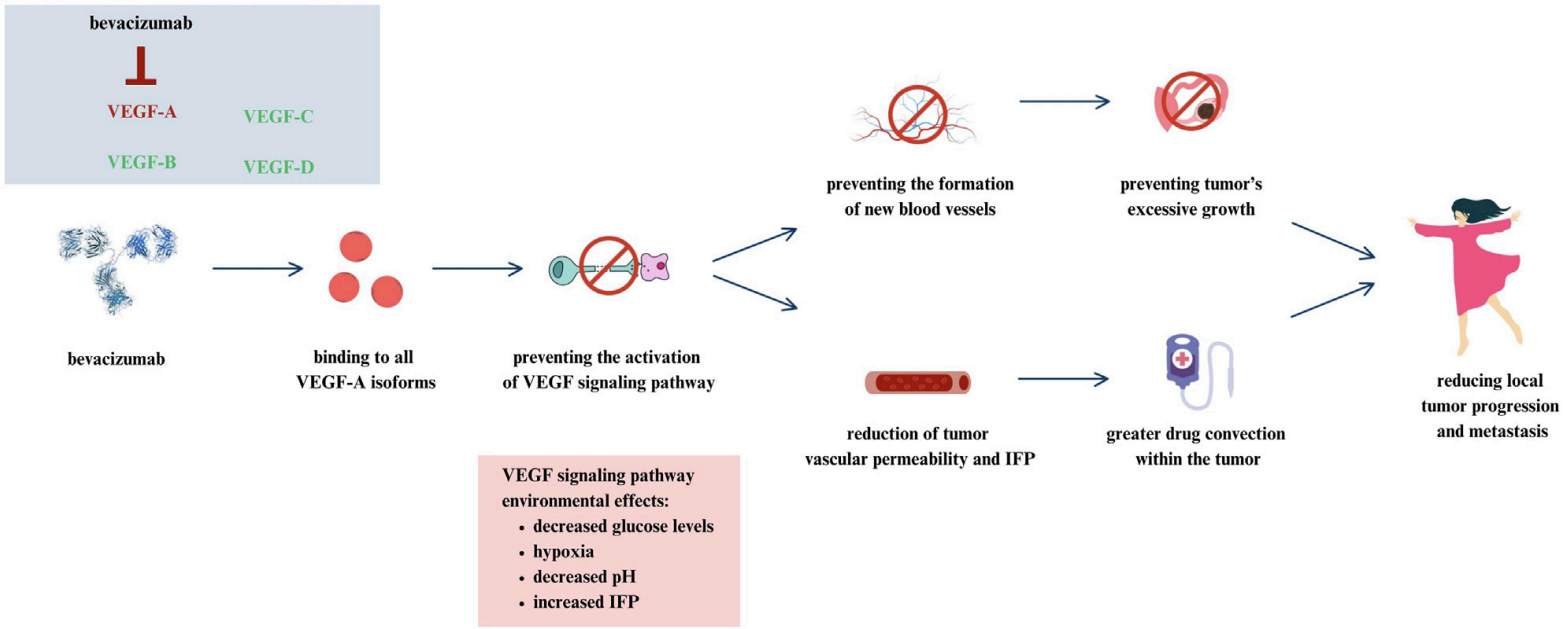
Mechanism of action for bevacizumab in ovarian cancer (Żak et al., 2024).

Bevacizumab is an anti-VEGF antibody whose mechanism of action is based on the inhibition of angiogenesis, thus depriving the tumor of the ability to grow and develop. Bevacizumab for the treatment of stage III or IV epithelial ovarian cancer was approved by the EMA in 2011 and by the FDA in 2018. This medicine is used for 15 months. It is one of the first drugs whose therapy is based on targeting the tumor microenvironment (Nakai and Matsumura, 2022; Żak et al., 2024). The exact mechanism of action for bevacizumab in the treatment of ovarian cancer is shown in Figure 2.

Why is bevacizumab so special? This is the first targeted therapy in almost 40 years to treat advanced ovarian cancer (Garcia et al., 2020). In this context, the results of the GOG-218 study are important. This is a double-blind, placebo-controlled phase III study. In this study, 1,873 women were included in the group receiving chemotherapy (paclitaxel and carboplatin) with placebo in cycles 2 to 22, in the group receiving bevacizumab at a dose of 15 mg/kg body weight. in cycles 2 to 6 and placebo from 7 to 22 together with chemotherapy, and to the group receiving chemotherapy with bevacizumab in cycles 2 to 22. The median PFS in these groups was 10.3 months, 11.2 months and 14.1 months (Burger et al., 2011).

Moreover, the results of the ICON-7 study seem interesting. It was an international, open-label, randomized phase III trial. 1,528 women were assigned to the group receiving chemotherapy alone or chemotherapy plus bevacizumab at a dose of 7.5 mg/kg body weight. every 3 weeks intravenously. The median OS in these groups was 44.6 and 45.4 months, and the median PFS was 34.5 and 36.3 months. This study confirmed the effectiveness of bevacizumab in the primary treatment of patients with ovarian cancer (Oza et al., 2015).

Moreover, it turns out that bevacizumab in combination with carboplatin also prolongs PFS in patients. Results of PAOLA-1 - a randomized, double-blind phase III study, showed the benefits of using olaparib together with bevacizumab (15 mg/kg every 3 weeks for 15 months). The median OS was 56.5 months in patients treated with the combination of olaparib and bevacizumab and 51.5 months in the group receiving placebo plus bevacizumab. Interestingly, the 5-year OS was higher in patients with HRD-added ovarian cancer, as it amounted to 65.5%. In patients with HRD-negative ovarian cancer, it was 48.4% (Ray-Coquard et al., 2023). The results of the PAOLA-1 study indicate the need for biomarker testing in patients with ovarian cancer.

AGO-OVAR 17 BOOD/GINECO OV118/ENGOT Ov15 is an open-label, randomized phase III study. In 927 patients qualified for the study, a comparison of treatment with bevacizumab and chemotherapy was assessed. First, the patients were treated with cytoreductive surgery with 6 cycles of chemotherapy with paclitaxel and bevacizumab at a dose of 15 mg/kg body weight. 1 time every 3 weeks. The median PFS was 26.0 months in patients treated with extended bevacizumab and 24.2 months in patients treated standardly. No differences were observed in median OS. Therefore, the study did not show that long-term bevacizumab treatment had a significant impact on PFS or OS (Pfisterer et al., 2023).

The results of the study, which aimed to evaluate the use of the combination of bevacizumab and mirvetuximab soravtensine in patients with platinum-resistant ovarian cancer, are interesting. 94 patients were enrolled and received bevacizumab at a dose of 15 mg/kg body weight. intravenously once every 3 weeks and mirvetuximab soravtenise. 59% of patients were previously treated with bevacizumab, 52% with ≥3 therapies, and 27% with PARP inhibitor therapy. The median PFS was 8.2 months in this study. Results were promising regardless of folate receptor alpha (FRAα) expression or prior treatment (Gilbert et al., 2023).

The clinical trials describing the efficacy of bevacizumab in patients with ovarian cancer are presented in Table 1.

**TABLE 1:** Clinical trials with bevacizumab in ovarian cancer.

Name of the study	Year of the study	Phase of the study	Research group	Dose of bevacizumab	Results
GOG-218 (Burger et al., 2011)	2011	III	1873, (control therapy n = 625, bevacizumab-initiation therapy n = 625, bevacizumab-throughout therapy n = 623)	Bevacizumab-initiation: chemotherapy + bevacizumab (15 mg/kg), cycles 2–6, placebo, cycles 7–22; bevacizumab-throughout: chemotherapy + bevacizumab, cycles: 2–22	Control group, bevacizumab-initiation group, bevacizumab-throughout group: PFS 10.3, 11.2, 14.1 months; OS 39.3, 38.7, 39.7 months, respectively
ICON-7 (Oza et al., 2015)	2015	III	1,528, (standard chemotherapy n = 764, chemotherapy plus bevacizumab n = 764)	7.5 mg/kg every 3 weeks	Standard chemotherapy group, chemotherapy plus bevacizumab group: PFS 34.5, 36.3 months; OS 44.6, 45.4 months, respectively
PAOLA-1 (Ray-Coquard et al., 2023)	2023	III	806, (olaparib plus bevacizumab n = 537, placebo plus bevacizumab n = 269)	15 mg/kg every 3 weeks for 15 months	Olaparib plus bevacizumab, placebo plus bevacizumab: OS 56.5, 51.6 months, respectively
AGO-OVAR 17 BOOD/GINECO OV118/ENGOT Ov15 (Pfisterer et al., 2023)	2023	III	927, (standard chemotherapy plus bevacizumab for 15 months n = 464, standard chemotherapy plus bevacizumab for 30 months n = 463)	15 mg/kg once every 3 weeks for 15 or 30 months	Standard chemotherapy plus bevacizumab for 15 months, standard chemotherapy plus bevacizumab for 30 months: PFS 24.2, 26.0 months; OS 60.4, 60.8 months, respectively
Gilbert et al. (Gilbert et al., 2023)	2023	Ib/II	94, (combination treatment with mirvetuximab soravtansine and bevacizumab n = 94)	15 mg/kg	PFS: 8.2 months, DOR: 9.7 months

PFS, progression-free survival; OS, overall survival; DOR, duration of response.

### 3.2 Cediranib

Cediranib is another anti-aniogenic drug that is a multikinase inhibitor acting against VEGF receptor 3 (VEGFR1-VEGFR3). So far, the beneficial use of this inhibitor has been described in ovarian cancer, lung cancer, glioblastoma multiforme and kidney cancer (Matulonis et al., 2009; Goss et al., 2010; Batchelor et al., 2010; Mulders et al., 2012). So far, the results of studies have shown an increase in progression-free survival and overall survival in patients with ovarian cancer as a result of the use of cediranib in combination with chemotherapy and PARP inhibitors (Liu et al., 2019).

In 2021, the results of the ICON6 study were published - a three-arm, double-blind, placebo-controlled randomized trial, the aim of which was to examine the effectiveness of cediranib in 456 patients with recurrent platinum-sensitive ovarian cancer. Patients were randomly assigned to three arms in a 2:3:3 ratio. Arm A consisted of patients receiving chemotherapy with oral placebo and continuing supportive care. Patients in arm B received daily oral cediranib during chemotherapy and then received placebo during chemotherapy. In turn, patients in arm C received cediranib during chemotherapy and continued to take it as maintenance therapy. The daily dose of cediranib was 20 mg. The median follow-up period was 7 years for arm A and 83.7 months for arm C. The median survival in arm A was 19.9 months and in arm C 27.3 months. Moreover, in arm C, the time to death over 6 years was increased by an average of 4.8 months compared to arm A. The median survival time in arm B was similar to the results in arm C and amounted to 26.6 months. The reasons for discontinuing the drug in patients were symptoms such as diarrhea, neutropenia, voice changes or hypertension (Ledermann et al., 2021). Despite an increase in progression-free survival, cediranib caused toxic effects.

In platinum-sensitive ovarian cancer, there is evidence of beneficial effects when antiangiogenic agents are used synergistically with PARP inhibitors. Preclinical study by Kaplan et al. from 2019 showed that due to the ability of cediranib to increase sensitivity to PARP inhibition, it may be beneficial to combine it with olaparib in patients with ovarian cancer (Kaplan et al., 2019). Study NRG-GY004 is an open-label, randomized, phase 3 study designed to evaluate the activity of olaparib or olaparib plus cediranib compared with platinum chemotherapy in 565 patients with ovarian cancer. The median PFS was 10.3 months for platinum-based chemotherapy, 8.2 months for olaparib, and 10.4 months for olaparib plus cediranib (Liu et al., 2019). Although the median PFS in the group of patients using olaparib with cediranib was not significantly higher than in the group of patients using chemotherapy, the results of this study should be a reason to conduct further studies related to the use of non-chemotherapy-based therapy in patients, which may prevent potential toxicity. chemotherapy. This seems extremely important considering that in this study 20 patients withdrew from the study after being assigned to chemotherapy. Perhaps this was due to fear of the side effects of this therapy, which further emphasizes the need to continue looking for alternative methods of treating ovarian cancer.

In summary, cediranib may increase the median PFS in female patients, but due to severe toxicity and the small number of studies, it seems important to further investigate its effectiveness in patients.

The clinical trials describing the efficacy of cediranib in patients with ovarian cancer are presented in Table 2.

**TABLE 2:** Clinical trials with cediranib in ovarian cancer.

Name of the study	Year of the study	Phase of the study	Research group	Dose of cediranib	Results
ICON6 (Ledermann et al., 2021)	2021	III	456, (chemotherapy plus placebo n = 118, chemotherapy plus cediranib with placebo maintenance n = 174, chemotherapy plus cediranib with cediranib maintenance n = 164)	Daily dose 20 mg	Chemotherapy plus placebo, chemotherapy plus cediranib with placebo maintenance, chemotherapy plus cediranib with cediranib maintenance: OS 19.9, 26.6, 27.3 months, respectively
NRG-GY004 (Liu et al., 2019)	2022	III	565, (platinum-based chemotherapy n = 187, olaparib alone n = 189, olaparib plus cediranib)	30 mg once daily	Platinum-based chemotherapy, olaparib alone, olaparib plus cediranib: PFS 10.3, 8.2, 10.4 months; OS 31.2, 29.2, 31.3, respectively

PFS, progression-free survival; OS, overall survival.

### 3.3 Nintedanib

Nintedanib is another angiogenesis inhibitor, acting on VEGF 1-3, FGFR 1-3 and PDGFR α and β receptors, which has a shorter half-life of 10–15 h than bevacizumab (14–21 days). By targeting so many receptors, studies have demonstrated antitumor activity of nintedanib, as well as efficacy with docetaxel in patients with locally advanced and metastatic non-small cell lung cancer (Khalique and Banerjee, 2017). For this reason, over the last decade, further studies have been carried out to assess the effectiveness of nintedanib in the treatment of patients with ovarian cancer (Khalique and Banerjee, 2017; Wind et al., 2019).

AGO-OVAR 12 is a randomized phase III trial, the final results of which were presented in 2020. The study was designed to compare the effectiveness of administering nintedanib with carboplatin and paclitaxel in a group of 911 patients with a placebo group (455 patients) who received carboplatin and paclitaxel. Median follow-up was 60.9 months. The median OS was 62.0 months in the nintedanib group and 62.8 months in the placebo group. The median PFS for these patients was 17.6 and 16.6 months, respectively. The most common side effects were diarrhea (78% of patients taking nintedanib vs 26% of patients taking placebo), nausea (65% vs 53%), and alopecia (58% vs 62% (Ray Coquard et al., 2020). The results of this study did not demonstrate that adding nintedanib to chemotherapy contributed to improved OS. Improved OS was observed in patients with peritoneal disease/ascites, which may be due to M1-polarized macrophages, which have been reported to be associated with ascites (Madeddu et al., 2018).

In 2020, the results of METRO-BIBF were published - a randomized, placebo-controlled study aimed at examining the effectiveness and safety of the combination of nintedanib with oral cyclophosphamide in patients with recurrent ovarian cancer. To our knowledge, this is the first study to analyze the effectiveness of this therapy in patients treated early with other intensive methods. Patients received oral cyclophosphamide 100 mg once daily and were randomized 1:1 to also receive placebo (n = 55) or nintedanib (n = 59). 35 patients were previously treated with bevacizumab and 55 patients were previously treated with ≥5 cycles of chemotherapy. The median OS was 6.8 months for patients in the nintedanib group and 6.4 months for patients in the placebo group. In turn, the median PFS was 2.9 months for patients taking nintedanib and 2.6 months for patients taking placebo. Moreover, in the study, patients took 100 mg of cyclophosphamide, whereas in other studies the dose of cyclophosphamide was 50 mg daily. The most common side effects in patients are lymphopenia, neutropenia, diarrhea, vomiting and fatigue. Toxicity was 10% lower in patients taking cyclophosphamide alone than in patients taking cyclophosphamide plus nintedanib (Hall et al., 2020). The study did not show that nintedanib improved treatment outcomes in patients taking cyclophosphamide.

The CHIVA study is a double-blind randomized phase II study, the results of which were presented in January 2023. The aim of the study was to determine the effectiveness of nintedanib with neoadjuvant chemotherapy (NACT) in patients after interval debulking surgery (IDS) with advanced ovarian cancer. A total of 188 patients with newly diagnosed ovarian cancer, FIGO stage IIIC/IV, who were not eligible for surgical treatment, were included in the study. Patients received chemotherapy with carboplatin AUC plus paclitaxel at a dose of 175 mg/m2. every 21 days for three to four cycles before and two to three cycles after IDS (up to 8 cycles). 124 patients also received nintedanib 200 mg and 64 patients received placebo twice daily on days 2–21 every 3 weeks during NACT and thereafter as maintenance treatment for approximately 2 years. The median PFS in patients taking nintedanib was 14.14, while the median in patients taking placebo was 16.8 months. The median OS was 37.3 and 44.1 months, respectively. Moreover, 92% of patients in the nintedanib group experienced side effects such as widespread hematological or gastrointestinal events, compared to the placebo group, where these symptoms occurred in 69% of patients (Ferron et al., 2023). The study results showed a clear lack of improvement in efficacy when nintedanib was added to NACT. This is actually consistent with the results of other studies that also evaluated the effect of adding antiangiogenic therapy to NACT (Garcia Garcia et al., 2019). A limitation of this study is that the study included inoperable patients with multiple comorbidities and deteriorated condition, which may have influenced the final treatment outcome. It also seems important to focus on the toxicity of chemotherapy in subsequent studies.

The results of randomized trials did not show that the use of nintedanib led to a significant increase in median PFS and OS in patients with ovarian cancer. Moreover, in each of the described studies, patients experienced significant side effects related to its toxicity. Therefore, caution is required when adding antiangiogenic therapy to chemotherapy in the neoadjuvant treatment.

The clinical trials describing the efficacy of nintedanib in patients with ovarian cancer are presented in Table 3.

**TABLE 3:** Clinical trials with nintedanib in ovarian cancer.

Name of the study	Year of the study	Phase of the study	Research group	Dose of nintedanib	Results
AGO-OVAR 12 (Ray Coquard et al., 2020)	2020	III	1,366, (nintedanib group n = 911, placebo group n = 455)	200 mg twice daily on days 2–21 every 3 weeks for up to 120 weeks	Nintedanib group, placebo group: PFS 17.6, 16.6 months; OS 62.0, 62.8 months, respectively
METRO-BIBF (Hall et al., 2020)	2020	II	117, (oral cyclophosphamide plus nintedanib group n = 59, oral cyclophosphamide plus placebo group n = 58)	Starting dose was 200 mg twice daily	Oral cyclophosphamide plus nintedanib group, oral cyclophosphamide plus placebo group: PFS 2.9, 2.6 months; OS 6.8, 6.4 months, respectively
CHIVA (Ferron et al., 2023)	2023	II	188, (nintedanib group n = 124, placebo group n = 64)	200 mg on days 2–21 every 3 weeks during NACT and thereafter as maintenance treatment for approximately 2 years	Nintedanib group, placebo group: PFS 14.4, 16.8 months; OS 37.3, 44.1 months, respectively

NACT, neoadjuvant chemotherapy; PFS, progression-free survival; OS, overall survival.

### 3.4 Pazopanib

Another option in the treatment of ovarian cancer seems to be pazopanib. It is a small molecule inhibitor of VEGFR 1-3, c-Kit and platelet-derived growth factor receptor α and β (PDGFRA and PDGFRB) (Du Bois et al., 2014). It is true that there are a limited number of studies assessing the effect of pazopanib on the treatment of patients with ovarian cancer, and the current ones indicate quite high toxicity of the therapy in the form of side effects such as diarrhea (Friedlander et al., 2018). Therefore, the latest studies are based on the use of pazopanib at a reduced dose, with the aim of reducing the likelihood of toxicity in patients.

In 2020, the results of NCT01610206 were published - an open-label, randomized, multi-site, phase 2 study that assessed the effectiveness of adding pazopanib to gemcitabine in 148 patients with platinum-resistant or sensitive ovarian cancer after ≤3 previous lines of chemotherapy. Patients were randomized 1:1 to receive gemcitabine 1,000 mg/m2 weekly on days 1, 2, and 8 intravenously for up to 21 days with or without pazopanib 800 mg orally daily. The median PFS was 2.9 in patients receiving gemcitabine alone and 5.3 months in patients receiving combination therapy with pazopanib. A significantly greater number of side effects such as anemia, neutropenia, thrombocytopenia, fatigue, elevated AST and hypertension occurred in the group of patients treated with combination therapy. Moreover, 14% of those treated with gemcitabine alone and 40% of those treated with gemcitabine plus pazopanib discontinued participation in the study due to side effects such as neutropenia, fatigue, or hepatotoxicity (Duska et al., 2020). Although the study showed an improvement in median PFS in patients using the combination therapy, a large number of side effects were also reported. Moreover, the limitation of this study is definitely the unselected patient population. Moreover, in this study, the high dose of pazopanib of 800 mg orally daily may have caused such significant toxicity, supporting the need for further studies using lower doses of pazopanib.

Sharma et al. in a 2021 study aimed to evaluate the use of oral metronomic therapy in 75 patients with platinum- or treatment-resistant epithelial ovarian cancer. 38 patients in group A received etoposide 50 mg from days 1–14 and cyclophosphamide 50 mg from days 1–28 every 4 weeks. In turn, 37 patients from group B received the same treatment in combination with pazopanib at a dose of 400 mg once daily. The median PFS was 3.4 months in patients in group A and 5.1 months in patients in group B. The median OS in group A was 11.2 months, and in group B it was “not achieved”. Side effects occurred in 19 patients from group A and 22 patients from group B. Only in patients from group B, side effects such as hypertension (5.4%) and increased liver enzymes (5.4%) were recorded (Sharma et al., 2021). The study results showed an increase in the median PFS and OS in patients treated with pazopanib with cyclophosphamide and etoposide in combination therapy, however, the limitation of this study is the definitely small number of qualified patients and the fact that it was a single-center study.

In 2022, the final results of the randomized phase II TAPAZ trial were published, the aim of which was to determine the effectiveness of the combination of paclitaxel and pazopanib at a lower dose than in other studies. The study enrolled 116 patients with recurrent ovarian cancer who had previously been treated with bevacizumab. 79 patients were treated with paclitaxel 65 mg/m2 on days 1, 8 and 15 intravenously together with pazopanib 600 mg/day orally. 37 patients were treated with intravenous paclitaxel alone at a dose of 80 mg/m4 on days 1, 8 and 15 every 28 days. The median PFS was 4 months in the combination group and 68% in the paclitaxel alone group. In turn, the median OS in these groups was 13.6 and 12.9 months, respectively. 47% of patients in the paclitaxel plus pazopanib group discontinued treatment, compared to 11% in the paclitaxel alone group. Patients in the group receiving combination therapy were more likely to experience side effects such as hypertension, diarrhea, anorexia, proteinuria and thrombocytopenia than in patients using paclitaxel alone. Moreover, there was one death due to gastrointestinal perforation and 1 death due to pulmonary embolism, which may have been related to the use of pazopanib (Joly et al., 2022). The results of the TAPAZ trial not only showed no improvement in median or OS in patients treated with pazopanib, but also showed an increased risk of adverse events with this therapy. The results of this study appear to be similar to the CHIVA trial evaluating the use of nintedanib on paclitaxel, which we reported above (Ferron et al., 2023). Both of these studies did not show that the addition of a given angiogenesis inhibitor had a beneficial effect on PFS and OS, and in fact showed an increased likelihood of side effects (Joly et al., 2022; Ferron et al., 2023).

The PAZOFOS study also seems worth mentioning. This is a phase 1b and randomized phase 2 trial that assessed the effectiveness of pazopanib with fosbretabulin in patients with recurrent epithelial ovarian cancer with a platinum-free interval (PFI) of 3–12 months. To our knowledge, this is the first study to evaluate fosbretabulin with an angiogenesis inhibitor. In phase 1b, 12 patients received pazopanib at a dose of 600 mg once daily and fosbretabulin at a dose of 54 mg/m2 on days 1, 8 and 15 every 28 days (dose level 1), pazopanib at a dose of 800 mg once daily and fosbretabulin 54 mg/m2 on days 1, 8 and 15 every 28 days (dose level 2), and pazopanib at a dose of 800 mg once daily and fosbretabulin at a dose of 60 mg/m2 on days 1,8 and 15 every 28 days. In turn, in phase II of the study, patients were assigned to two groups. 10 patients received pazopanib at a dose of 600 mg once daily and fosbretabulin at a dose of 54 mg/m2 on days 1, 8 and 15 every 28 days. Eleven patients received pazopanib 800 mg once daily every 28 days until disease progression or adverse events occurred. Adverse events in phase 1B included hypertension, neutropenia, fatigue and vomiting. The median PFS in phase II was 7.6 months in patients receiving the combination of pazopanib and fosbretabulin and 3.7 months in patients receiving pazopanib alone (Morgan et al., 2020). The study results showed that combined treatment with pazopanib and fosbretabulin not only improved the PFS result in patients, but also caused significant cardiac toxicity in the form of increased troponin levels and left ventricular dysfunction in 2 patients. Future research must therefore determine which of these substances is responsible for these side effects.

Although the research results seem to be quite promising in patients with platinum-resistant ovarian cancer, the statistics regarding its toxicity and side effects seem disturbing. Before starting phase III trials or new studies, it is necessary for physicians to better and more effectively recognize and mitigate the adverse effects of pazopanib therapy, especially hypertension. Moreover, in order to minimize the risk of side effects, it may be necessary in the future to identify those patients who benefited most from this therapy, which highlights the role of biomarkers.

The clinical trials describing the efficacy of pazopanib in patients with ovarian cancer are presented in Table 4.

**TABLE 4:** Clinical trials with pazopanib in ovarian cancer.

Name of the study	Year of the study	Phase of the study	Research group	Dose of pazopanib	Results
NCT01610206 (Duska et al., 2020)	2020	II	148, (gemcitabine alone group n = 73, gemcitabine plus pazopanib group n = 75)	800 mg orally daily	Gemcitabine alone group, gemcitabine plus pazopanib group: PFS 2.9, 5.3 months; OS 1.3, 1.1 years, respectively
CTRI/2017/10/010219 (Sharma et al., 2021)	2021	II	75, (etoposide and cyclophosphamide group n = 38, etoposide and cyclophosphamide plus pazopanib group n = 37)	400 mg once daily	Etoposide and cyclophosphamide group, etoposide and cyclophosphamide plus pazopanib group: PFS 3.4, 5.1 months; OS 11.2 months, not reached, respectively
TAPAZ (Joly et al., 2022)	2022	II	116, (paclitaxel plus pazopanib group n = 79, paclitaxel only group n = 37)	600 mg/day orally	Paclitaxel plus pazopanib group, paclitaxel only group: PFS 4.9, 5.8 moths; OS 13.6, 12.9 months, respectively
PAZOFOS (Morgan et al., 2020)	2020	Ib/II	Ib: 12, (pazopanib plus fosbretabulin group n = 12) II: 21, (pazopanib only group n = 10, pazopanib plus fosbretabulin group n = 11)	Ib: 600 mg once daily (level 1), 800 mg once daily (level 2) II: 800 mg once daily (pazopanib only group n = 10), 600 mg once daily (pazopanib plus fosbretabulin group n = 11)	II. Pazopanib only group, pazopanib plus fosbretabulin group: PFS 3.7, 7.6 months, OS 8.4 months, not reached, respectively

PFS, progression-free survival; OS, overall survival.

## 4 PARP inhibitors

Poly(ADP-ribose) polymerase (PARP) inhibitors are another option for targeted therapy in the treatment of ovarian cancer. These anticancer drugs bind PARP1 and PARP2, which in turn are involved in DNA repair (Zaremba and Curtin, 2007). Inhibited PARP proteins cannot dissociate from DNA, which makes them unable to coordinate repair at other sites of DNA damage. The concept of synthetic lethality is important, which means that two genetic mutations occurring separately are not harmful (fda.gov, 2022; Jones et al., 2015; Bryant et al., 2005; Farmer et al., 2005; Liu et al., 2014). However, they can cause cell death when combined. When inhibited, PARP, BRCA and other proteins of the homologous recombination repair pathway repair DNA (Saleh-Gohari et al., 2005; Yap et al., 2011). Homologous recombination deficiency (HRD) will result from inactivation of BRCA1 or BRCA2 in the cancer cell. This mechanism shows that PARP inhibitors lead to DNA damage and thus the death of cancer cells, which is used in solid tumors. The first PARP inhibitors approved by the US FDA are olaparib, niraparib, and rucaparib, which are maintenance therapy for patients with ovarian cancer (O Malley et al., 2023). In this chapter, we analyzed the latest clinical trials on PARP inhibitors and discussed future challenges and goals for this therapy.

### 4.1 Olaparib

Olaparib (LYNPARZA^®^, AstraZeneca Pharmaceuticals LP) - an inhibitor of human PARP-1, PARP-2 and PARP3, is the first PARP inhibitor approved by the FDA in 2014 for the treatment of metastatic ovarian cancer (Zhou et al., 2019; Arora et al., 2021). Olaparib is used in women with advanced-stage epithelial ovarian cancer, fallopian tube cancer, or primary peritoneal cancer when first germline or somatic BRCA1/2 mutations are present or recurrent platinum-sensitive ovarian cancer after complete or partial response to platinum-based chemotherapy (Frampton, 2015; Heo and Dhillon, 2018). The first study to demonstrate the effectiveness of olaparib in ovarian cancer is Study 19, which evaluated the drug *versus* placebo in 136 patients with recurrent, high-grade, sensitive, serous ovarian cancer. to platinum. In patients taking olaparib, the median PFS was 11.2 months, and in the placebo group, the median was 4.3 months. No major difference was observed between the median OS in both groups. Regarding grade 1 and 2 adverse events, patients reported mainly fatigue, nausea, vomiting, taste change and anorexia. Grade ≥3 adverse events were reported more frequently in patients in the olaparib group (40%) than in the placebo group (22%) and included nausea, fatigue, neutropenia, and anemia (Ledermann et al., 2014). The results of this study clearly demonstrated that olaparib is an effective therapy in patients with platinum-sensitive recurrent BRCA-mutated serous ovarian cancer. It was the results of this study that contributed to the approval of this drug by the FDA.

The CLIO/BGOG study, which included 160 patients, compared olaparib monotherapy with chemotherapy in patients with platinum-sensitive or resistant ovarian cancer without BRCA mutations or recurrence. 107 patients were assigned to the olaparib group and 53 to the chemotherapy group, including 89 and 49 patients in these groups who did not have a BRCA mutation. The clinical benefit rate (CBR) was achieved by 58 patients from the olaparib group and 30 patients from the chemotherapy group. The median PFS was 4.8 and 5.7 months in these groups, and the median OS was 12.5 and 14.4 months (Vanderstichele et al., 2022). The study results showed similar effectiveness of treatment with both olaparib and chemotherapy. Moreover, these results are valuable for the treatment of patients with ovarian cancer that is sensitive or resistant to standard chemotherapy treatment. It also seems important that this study assessed the effect of olaparib treatment in patients without BRCA mutations.

SOLO2/ENGOT Ov-21 is a randomized phase III trial that evaluated olaparib in women with recurrent platinum-sensitive BRCA1/2 mutation-positive ovarian cancer (BRCA) after response to platinum-based chemotherapy. A *post hoc* analysis of this study was performed in 2023. 147 patients were assigned to the group receiving olaparib (53%) in the form of tablets at a dose of 300 mg twice daily or the placebo group (47%). In the olaparib group, 24 and 54 patients received platinum-free chemotherapy and platinum-containing chemotherapy, respectively, while in the placebo group, the numbers were exactly 27 and 42 patients. Median OS was 51.1 months in patients taking olaparib compared with 38.8 months in patients in the placebo group, and median PFS was 18.4 months in the placebo group (not achieved for the olaparib group). Time to second subsequent treatment (TTSP) was 12.1 months in the placebo group and 6.9 months in the olaparib group. The results of this question lead to reflection on what treatment would be most optimal in patients with early relapse after treatment with a PARP inhibitor (Poveda et al., 2021).

PAOLA-1/ENGOT-ov25 is a double-blind, phase III trial, the aim of which was to evaluate maintenance treatment with olaparib together with bevacizumab in patients diagnosed with ovarian cancer with response after first-line chemotherapy in the form of platinum compounds with bevacizumab. The final analysis of the study results were published in 2023. 535/537 patients received olaparib 300 mg twice daily for up to 24 months in combination with bevacizumab 15 mg/kg every 3 weeks for a total of 15 months and 267/269 patients received placebo in combination with bevacizumab. The final median OS was 56.5 months in the olaparib group and 51.6 months in the placebo group. Moreover, OS was longer in patients with positive HRD (65.5% vs 48.4%). The median PFS in these groups was 46.1% and 19.2%. In the group receiving olaparib, 9 cases of myelodysplastic syndromes, acute myeloid leukemia, and amyloidosis were recorded, and in the group receiving placebo, 6 cases. New primary malignancies occurred in 22 and 8 patients respectively (olaparib vs placebo), and pneumonia occurred in 7 and 2 patients respectively (Ray-Coquard et al., 2023). This study did not include a group treated with olaparib as monotherapy, which makes it difficult to determine the exact effect of olaparib and bevacizumab *versus* olaparib alone. Therefore, it is necessary to conduct further studies that will assess the impact of both combination therapy and monotherapy with a PARP inhibitor. Moreover, this is another study whose results clearly emphasize the importance of conducting research on biomarker tests, which will help to better and more precisely determine the groups of patients who will respond best to treatment with PARP inhibitors. It is important to analyze the median OS in patients depending on the location or type of BRCA mutation. Taking into account the fact that patients with HRD-positive disease responded best to treatment with PARP inhibitors, a question should be asked about possible treatment options for patients with HRD-negative disease.

In 2023, the results of the double-blind phase III trial SOLO1/GOG 3004 were published after 7 years of follow-up, which included the treatment of patients with newly diagnosed advanced ovarian cancer and BRCA mutation after platinum-based chemotherapy with olaparib. Patients were randomized to olaparib tablets 300 mg twice daily (n = 260) or placebo (n = 131). After 7 years, 67.0% of patients in the olaparib group and 46.5% of patients in the placebo group were alive. The median follow-up in this study was approximately 88 months, which, to our knowledge, is the longest follow-up of any PARP inhibitor in ovarian cancer (DiSilvestro et al., 2023). Moreover, the study results showed improved OS in patients with newly diagnosed ovarian cancer treated supportively with olaparib.

The use of olaparib in patients with ovarian cancer represents a significant progress in treatment. In the PAOLA-1 (Ray-Coquard et al., 2023) trial, patient selection was not driven by BRCAm status compared to the SOLO-1 (DiSilvestro et al., 2023) trial, which was based on patients with a germline BRCA mutation. In contrast, the CLIO/BEGOG trial also focused on patients without BRCA mutations (Vanderstichele et al., 2022). It is hypothesized that a germline or somatic BRCA mutation causes HRR deficiency, leading to sensitivity to PARP inhibition (Arora et al., 2021).

The clinical trials describing the efficacy of olaparib in patients with ovarian cancer are presented in Table 5.

**TABLE 5:** Clinical trials with olaparib in ovarian cancer.

Name of the study	Year of the study	Phase of the study	Research group	Dose of olaparib	Results
NCT00753545 (Ledermann et al., 2014)	2014	II	265, (olaparib group n = 136, placebo group n = 129)	400 mg twice daily, capsules	Olaparib group, placebo group: PFS 11.2, 4.3 months; OS 37.1, 37.6 months, respectively
CLIO/BGOG-ov10 (Vanderstichele et al., 2022)	2022	II	160, (olaparib only group n = 107, standard chemotherapy group n = 53)	Starting dose of 300 mg (2 × 150 mg tablets)	Olaparib only group, standard chemotherapy group: PFS 4.8, 5.7 months; OS 12.5, 14.4 months, respectively
SOLO2/ENGOT Ov-21 (Poveda et al., 2021)	2023	III	295 (olaparib only group n = 195, standard chemotherapy group n = 990)	300 mg in two 150 mg tablets, twice daily	Olaparib only group, standard chemotherapy group: PFS not achieved, 18.4 months; OS 51.1, 38.8 months, respectively
PAOLA-1/ENGOT-ov25 (Ray-Coquard et al., 2023)	2023	III	806, (olaparib plus bevacizumab n = 537, placebo plus bevacizumab n = 269)	300 mg twice daily	Olaparib plus bevacizumab, placebo plus bevacizumab: OS 56.5, 51.6 months, respectively
SOLO1/GOG 3004 (DiSilvestro et al., 2023)	2023	III	391, (olaparib group n = 260, placebo group n = 130)	300 mg twice daily, tablets	Olaparib group, placebo group: TFST 64.0, 15.1 months; OS not reached, 75.2 months, respectively

PFS, progression-free survival; OS, overall survival; TFST, time to first subsequent therapy or death.

### 4.2 Niraparib

Niraparib (MK4827) is an oral PARP-1 and PARP-2 inhibitor that causes cancer cell death with BRCA1 and BRCA2 deficient cell lines (AlHilli et al., 2016; Caruso et al., 2017) Already in 2012, Wang et al. described the increased effectiveness of radiotherapy in human lung and breast xenografts in combination with niraparib (Wang et al., 2012). Moreover, we find that in patients with recurrent platinum-sensitive ovarian cancer, niraparib improved median PFS regardless of BRCA mutation. Thanks to the results of the ENGOT-OV16/NOVA study on 553 patients, in 2017 the US FDA approved the use of niraparib in patients with relapsed ovarian cancer in the CR or PR phase with platinum-based chemotherapy. The results of this study showed not only a higher median PFS in patients with BRCA mutations (12.9 months in the niraparib group vs 3.8 months in the placebo group), but also in patients without mutations (6.0 months vs 3.9 months) (Mirza et al., 2016).

Although the main PARP inhibitors described in the literature for use in patients with ovarian cancer are olaparib and niraparib, it turns out that research on both of these inhibitors may be contradictory. The double-blind phase III NORA trial analyzed niraparib maintenance in patients with platinum-sensitive relapsed ovarian cancer. There were 177 patients in the niraparib group and 88 in the placebo group. 14 patients with a median weight of 82.5 kg received niraparib or placebo at a dose of 300 mg, and 235 patients with a median weight of 59.0 kg received a dose of 200 mg. The median PFS was 18.3 months in the niraparib group and 5.4 months in the placebo group (Wu et al., 2021). Furthermore, for patients taking niraparib, the median PFS was 11.1 for germline BRCA mutations and 3.9 months for germline BRCA negative patients, consistent with the results of the NOVA trial [Wu XH]. Median OS data is not yet mature. The most frequently reported adverse events were decreased neutrophil counts (20.3% of patients in the niraparib group vs 9.0% of patients in the placebo group) and anemia (14.7% vs 2.3%, respectively). The results of this study not only demonstrated the effectiveness of niraparib in recurrent platinum-sensitive ovarian cancer, but also its effectiveness regardless of the presence or absence of BRCA mutations. Moreover, to our knowledge, this is the first study that established an individual drug dosing regimen. The low number of adverse events may have been due to the fact that a large proportion of patients were initially treated with a lower dose of niraparib (200 mg daily) (Wu et al., 2021).

In 2023, 3.5 years of follow-up results of the randomized phase III trial PRIMA/ENGOT-OV26/GOG-3012 were published. The aim of the study was to evaluate the effectiveness of niraparib in patients with newly diagnosed ovarian cancer after achieving a complete (CR) or partial response (PR) to first-line platinum chemotherapy. The study included 487 patients in the niraparib group and 246 patients in the placebo group. The median INV-PFS was 24.5 months in the group of patients taking niraparib and 11.2 months in the placebo group (hazard ratio, 0.52; 95% confidence interval [CI], 0.40–0.68) in the HRd population and 18.8 and 8.2 months in the entire patient population. As for the OS, it was still immature. Adverse events mainly included thrombocytopenia (39.7%), anemia (31.6%), and neutropenia (21.3%) (González-Martín et al., 2023). The results of the *ad hoc* analysis of this study demonstrated the efficacy of niraparib in female patients. However, despite the favorable results of this study, it is not yet possible to evaluate the long-term role of niraparib due to the lack of accurate median OS data.

The results of a phase III randomized clinical trial were published in 2023. Li et al. demonstrated prolonged PFS in patients with newly diagnosed ovarian cancer regardless of biomarker status or residual disease with niraparib maintenance therapy. 255 patients were assigned to the niraparib group and 129 patients to the placebo group. Median PFS was 24.8 months in the niraparib group and 8.3 months in the placebo group in the intention-to-treat population HR, 0.45; 95% CI, 0.34–0.60; *p* < .001}. Moreover, increased median PFS was also demonstrated in patients without germline BRCA variants (19.3 vs. 8.3 months; HR, 0.48; 95% CI, 0.34–0.67) and in homologous recombination deficient (16.6 vs. 5.5 months; HR, 0.44; 95% CI, 0.32–0.61) (Li et al., 2023).

Considering the favorable results of niraparib treatment in patients, the results of studies on its use in combination therapies may also be important. NItCHE trial (MITO 33) is a phase III, multicenter trial, the preliminary results of which are expected to be presented in June 2024. The aim of the study is to evaluate therapy with niraparib plus dostarlimab compared to chemotherapy alone in eligible patients with recurrent ovarian cancer. for treatment with platinum derivatives (Musacchio et al., 2021).

The clinical trials describing the efficacy of niraparib in patients with ovarian cancer are presented in Table 6.

**TABLE 6:** Clinical trials with niraparib in ovarian cancer.

Name of the study	Year of the study	Phase of the study	Research group	Dose of niraparib	Results
ENGOT-OV16/NOVA (Mirza et al., 2016)	2016	III	553 patients: 203 patients in the gBRCA cohort (niraparib group n = 138, placebo group n = 65), 350 patients in the non-gBRCA cohort (niraparib group n = 234, placebo group n = 116)	300 mg once daily	Niraparib group, placebo group: gBRCA cohort - PFS 21.0, 5.5 months; non-gBRCA cohort - PFS 12.9, 3.8 months, respectively
NORA trial (NCT03705156) (Wu et al., 2021)	2021	III	265, (niraparib group n = 177, placebo group n = 88)	300 mg/day or 200 mg/day (depending on bodyweight and platelet count)	Niraparib group, placebo group: PFS 18.3, 5.4 months, TFST 16.7, 7.7 months, respectively
PRIMA/ENGOT-OV26/GOG-3012 (González-Martín et al., 2023)	2023	III	733, (niraparib group n = 487, placebo group n = 246)	300 mg/day or 200 mg/day (depending on bodyweight and platelet count)	Niraparib group, placebo group: overall population - PFS 13.8, 8.2 months; HRd population - PFS 24.5, 11.2 months; HRp population - 8.4, 5.4 months, respectively
Li et al. (Li et al., 2023)	2023	III	384, (niraparib group n = 255, placebo group n = 129)	300 mg/day or 200 mg/day (depending on bodyweight and platelet count)	Niraparib group, placebo group: PFS 24.8, 8.3 months, respectively

gBRCA, germline BRCA, mutation; PFS, progression-free survival; TFST, time to first subsequent therapy or death; HR, homologous recombination deficiency status (HRd, deficient; HRp, proficient or not determined).

### 4.3 Rucaparib

Rucaparib is another PARP-1/2/3 inhibitor that has been shown to be effective in the treatment of ovarian cancer (Drew et al., 2011; Coleman et al., 2017b; Yubero et al., 2022). Rucaparib was approved by the FDA in 2016 for the monotherapy treatment of patients with advanced ovarian cancer with BRCA mutations (germinal and/or somatic) who have had ≥2 cycles of chemotherapy (Syed, 2017). In turn, the results of the ARIEL3 trial supported the approval of rucaparib in 2019 by the European Medicines Agency (EMA) for the maintenance treatment of patients with platinum-sensitive recurrent ovarian cancer with a complete or partial response to platinum chemotherapy (Rubraca, 2022).

ARIEL3 is a double-blind, placebo-controlled, phase III trial in which 564 patients with platinum-sensitive ovarian cancer who received ≥2 cycles of platinum-based chemotherapy were randomized to rucaparib (n = 375) in 600 mg twice daily or placebo (n = 189). Median PFS was 8.2 months in the rucaparib group and 4.1 months in the placebo group (n = 224 vs. n = 113; HR 0.39, 95% CI 0.30 to 0.52, *p* < 0.0001) in patients with PFS 6 to ≤12 months and 13.6 months respectively and 5.6 months for patients with PFS >12 months. Moreover, PFS in the rucaparib group was 16.6 months (HR = 0.23, *p* < 0.0001) in the BRCA mutation group and 13.6 months (HR = 0.32, *p* < 0.0001) in the HRD group (including patients with BRCA mutations or wild/high LOH). Adverse events included anemia (18.8% in the rucaparib group and 0.5% in the placebo group) and increased alanine/aspartate aminitransferase activity (10.5% and 0%, respectively), indicating a fairly consistent and similar safety profile in patients in both groups (Clamp et al., 2021). The results of the ARIEL3 trial showed a benefit from rucaparib both in patients who had received 2 or more prior chemotherapy regimens and regardless of biomarker status.

ATHENA (NCT03522246) is a randomized, phase III trial evaluating rucaparib maintenance therapy in patients with stage III-IV advanced ovarian cancer who responded to first-line double platinum chemotherapy. 427 patients were assigned to rucaparib 600 mg twice daily and 111 patients were assigned to placebo. In the HRD population, the median PFS was 28.7 months in the rucaparib group and 11.3 months in the placebo group, in the intention-to-treat population it was 20.2 and 9.2 months, respectively, and in the HRD-negative population it was 12.1 and 9.1 months, respectively. The most common side effect was anemia (28.7% of patients in the study group and 0% in the placebo group) (Monk et al., 2022). The study results showed that, regardless of HRD or BRCA status, the median PFS was significantly higher in patients treated with rucaparib. Moreover, patients with HRD-negative tumors also benefited. This is important because patients with BRCA wild-type and HRD-negative tumors constituted 78.6% and 44.2% of the study population, respectively, which can only confirm the use of rucaparib in people who hypothetically benefit less from treatment with PARP inhibitors.

ARIEL4 is an open-label, randomized, controlled, phase 3 study comparing the efficacy of rucaparib *versus* platinum-based and non-platinum-based chemotherapy in 349 eligible patients with BRCA1/BRCA2 mutation-positive ovarian cancer who were receiving 2 or more chemotherapy regimens. This is the first study of its kind to compare any PARP inhibitor with or without platinum chemotherapy in patients with recurrent ovarian cancer and a BRCA1/BRCA2 mutation. 233 patients were assigned to receive rucaparib 600 mg twice daily orally and 116 patients to receive chemotherapy. Median PFS was 7.4 months in the rucaparib group and 5.7 months in the chemotherapy group HR 0·67 [95% CI 0·52–0·86]; *p* = 0·0017). The most common side effects were anemia or decreased hemoglobin, which is consistent with side effects in previous studies (Kristeleit et al., 2022). This is the first such study to show that patients with BRCA reversion mutations benefit less from treatment with rucaparib than patients without these mutations. Furthermore, it appears that in responding patients, the use of rucaparib may result in a durable response.

The clinical trials describing the efficacy of rucaparib in patients with ovarian cancer are presented in Table 7.

**TABLE 7:** Clinical trials with rucaparib in ovarian cancer.

Name of the study	Year of the study	Phase of the study	Research group	Dose of rucaparib	Results
ARIEL3 (Clamp et al., 2021)	2021	III	564, (rucaparib group n = 375, placebo group n = 189)	Rucaparib 600 mg twice daily	Rucaparib group, placebo group: progression-free interval 6–≤12 months - PFS 8.2, 4.1 months; progression-free interval >12 months - PFS 13.6, 5.6 months, respectively
ATHENA-MONO/GOG-3020/ENGOT-ov45 (Monk et al., 2022)	2022	III	538, (rucaparib group n = 427, placebo group n = 111)	Rucaparib 600 mg twice daily	Rucaparib group, placebo group: HRD population - PFS 28.7, 11.3 months; HRD-negative population - PFS 9.2, 9.1 months, respectively
ARIEL4 (Kristeleit et al., 2022)	2022	III	349, (rucaparib only group n = 233, chemotherapy group n = 116)	Rucaparib 600 mg twice daily	Rucaparib only group, chemotherapy group: PFS 7.4, 5.7 months, respectively

PFS, progression-free survival; HRD, homologous recombination deficiency.

## 5 Folate receptor alpha inhibitors

FRα is a glycoprotein anchored to glycosylphosphatidylinositol on the cell surface. Folic acid regulates the level of FRα expression, and its deficiency leads to increased FRα expression *in vivo* and *in vitro*. Moreover, FRα has the ability to participate in cell division, proliferation and tissue growth (Yang et al., 2007; Cheung et al., 2018). FRα is encoded by the FOLR1 gene and is expressed in breast and lung cancer, including on the plasma membrane of epithelial cells of the kidneys, placenta, uterus, cervix, and finally - ovary and fallopian tube (Bueno et al., 2001; Shi et al., 2009; O Shannessy et al., 2013). It turns out that FRα overexpression may occur in up to 90% of ovarian cancers (Kalli et al., 2008; Markert et al., 2008). In addition, FRα may be a biomarker for ovarian cancer because it can be detected in a soluble form in serum. Thanks to the possibility of assessing FRα protein expression using immunohistochemical staining, it is possible to qualify patients who may benefit from FRα-targeted therapy (Ebel et al., 2007). The first anti-FRα monoclonal antibody is farletuzumab (MORab003; Morphotek, Inc.), whose antitumor activity is based on the induction of antibody-dependent cellular cytotoxicity (ADCC), complement-dependent cytotoxicity (CDC), and inhibition of the Lyn kinase signaling pathway. However, a 2016 Phase III trial did not demonstrate that farletuzumab plus carboplatin and a taxane improved PFS outcomes in ovarian cancer patients (Vergote et al., 2016). Positive study results with mivetuximab soravtansine (MIRV) led to US FDA approval of MIRV for the treatment of platinum-resistant ovarian cancer in 2022 (Heo, 2023). Thus, an increasing number of studies are focusing on other anti-FRα monoclonal antibodies.

### 5.1 Mirvetuximab soravtansine

Mirvetuximab soravtansine (MIRV/Elagere/IMGN853) is an antibody-drug conjugate that consists of a humanized anti-FRα monoclonal antibody, a cleavable linker sulfo-SPDB and the cytotoxic maytansinoid effector molecule DM4 (Oroudjev et al., 2010; Ab et al., 2015). MIRV works by decomposing it to produce lysine-Nϵ-sulfo-SPDB-DM4. Subsequently, the maytansinoid derivatives DM4 and S-methyl-DM4 are formed by reduction and S-methylation of lysine-DM4. These substances suppress microtubule dynamics due to their strong anti-mitotic effect [Mai J]. The phase 1 IMGN853 trial aimed to establish the preliminary safety profile of MIRV in 44 patients. The study included 44 patients with FRα-positive solid tumors who received the drug at doses ranging from 0.15 to 7.0 mg/kg body weight. Of the patient cohort, 2 patients with epithelial ovarian cancer experienced clinical benefit which were confirmed tumor partial responses according to Response Evaluation Criteria in Solid Tumors 1.1. (Moore et al., 2017). The favorable results regarding the safety and tolerability of MIRV in ovarian cancer have become a reason to conduct further research on its use in patients with this cancer.

FORWARD II is a phase I study that aimed to evaluate the safety and tolerability of MIRV in combination with bevacizumab in 66 patients with platinum-resistant FRα-positive ovarian cancer. Patients were administered MIRV at a dose of 6 mg/kg along with bevacizumab at a dose of 15 mg/kg once every 3 weeks. The objective response rate (ORR) was 39%, including 5 complete and 21 partial responses. Median PFS was 6.9 months. The most common side effects were diarrhea, blurred vision, nausea and fatigue. The favorable results of the combination of MIRV and bevacizumab were encouraging to conduct further studies (O Malley et al., 2020).

SORAYA is a single-arm, phase II study that aimed to evaluate the safety and effectiveness of MIRV in 106 patients with platinum-resistant epithelial ovarian cancer. ORR was 32.4%, including 5 complete and 29 partial responses. Moreover, the ORR according to the investigator was 35.3% in patients with 1–2 treatments and 30.2% in patients with 3 treatments. The most common side effects included blurred vision, keratopathy, and nausea. For patients taking PARP inhibitors, the investigator-reported ORR was 38.0% and 27.5% for patients not taking PARP inhibitors (Matulonis et al., 2023).

FORWARD I is a randomized, open-label, phase III study designed to evaluate the efficacy and safety of MIRV compared with investigator’s choice of chemotherapy in 112 patients with ovarian cancer. 36 patients were assigned to the MIRV group. The median PFS in this group was 6.7 months (Moore et al., 2018). Given these encouraging results, a few years later the results of FORWARD I appeared, covering a larger population of 366 patients with platinum-resistant ovarian cancer. Patients who had previously received 1 to 3 therapies and had a medium or high level of FRα expression were qualified for the study. 243 patients received MIRV at a dose of 6 mg/kg and 109 received selected chemotherapy. The study results did not show a significant increase in the median PFS in the MIRV group (4.8 months) compared to the chemotherapy group (3.3 months) (Moore et al., 2021).

The clinical trials describing the efficacy of mirvetuximab soravtansine in patients with ovarian cancer are presented in Table 8.

**TABLE 8:** Clinical trials with mirvetuximab soravtansine in ovarian cancer.

Name of the study	Year of the study	Phase of the study	Research group	Dose of mirvetuximab soravtansine	Results
IMGN853 (Moore et al., 2017)	2017	I	44	Doses escalating from 0.15 to 7.0 mg/kg, once every 3 weeks	2 patients with epithelial ovarian cancer achieved confirmed tumor responses, according to Response Evaluation Criteria in Solid Tumors 1.1 - partial response
FORWARD II (O Malley et al., 2020)	2020	Ib	66	6 mg/kg, once every 3 weeks	PFS 6.9 months, ORR 39% (including 5 complete responses and 21 partial responses)
SORAYA (Matulonis et al., 2023)	2023	II	106	6 mg/kg, once every 3 weeks	PFS 4.3 months, OS 13.8
FORWARD I (Moore et al., 2021)	2021	III	352, (mirvetuximab soravtansine group n = 243, chemotherapy group n = 109)	6 mg/kg, once every 3 weeks	Mirvetuximab soravtansine group, chemotherapy group: PFS 4.8, 3.3 months, respectively

PFS, progression-free survival; ORR, objective response rate; OS, overall survival.

### 5.2 Farletuzumab

Farletuzumab (MORAb-003; Morphotek, Inc.) is the first humanized anti-FRα monoclonal antibody that has the ability to exert antitumor activity via antibody-dependent cytotoxicity (ADCC), complement-dependent cytotoxicity (CDC), tumor cell autophagy, and signaling pathway inhibition Lyn kinases (Ledermann et al., 2015; Sato and Itamochi, 2016).

In a phase I study already in 2010, the safety and good tolerability of farletuzumab was demonstrated in patients with platinum-refractory or platinum-resistant epithelial ovarian cancer. 25 patients received farletuzumab at a dose of 12.5–400 mg/m2 on days 1, 6, 15 and 22 of a 5-day cycle (Konner et al., 2010). Results from a 2013 study showed an increased response rate and duration of response among patients with platinum-sensitive ovarian cancer after treatment with fartletuzumab plus carboplatin and a taxane. Total or partial ORR was 75% (Armstrong et al., 2013).

In 2016, the results of a randomized, double-blind, placebo-controlled, phase III study were published, which assessed the effectiveness of farletuzumab in 1,100 patients with platinum-sensitive ovarian cancer. The median PFS was 9.0 months in the placebo group, 9.5 months in the farletuzumab 1.25 mg/kg group, and 9.7 months in the farletuzumab 2.5 mg/kg group. Side effects included those related to chemotherapy. Interestingly, the study showed that patients with higher exposure to farletuzumab and with CA-125 concentration no more than three times ULN had a better PFS result (Vergote et al., 2016). Therefore, although the study did not achieve final PFS, it likely identified those patients who may benefit from treatment with farletuzumab. Therefore, the aim of another randomized phase II trial was to determine the effectiveness of farletuzumab in improving PFS compared to placebo when added to standard chemotherapy in 214 patients with recurrent platinum-sensitive ovarian cancer with low CA-125 levels and at first recurrence. 142 patients received farletuzumab 5 mg/kg weekly with chemotherapy and 72 patients received chemotherapy with placebo. The study results did not show that the median PFS was significantly different between the farletuzumab and placebo groups. However, such study results may be due to the selection of a patient population with a lower CA-125 marker concentration, which correlates with a smaller disease volume and a potentially better immunological environment, which could affect the effectiveness of farletuzumab (Herzog et al., 2023).

The clinical trials describing the efficacy of farletuzumab in patients with ovarian cancer are presented in Table 9.

**TABLE 9:** Clinical trials with farletuzumab in ovarian cancer.

Name of the study	Year of the study	Phase of the study	Research group	Dose of farletuzumab	Results
Konner et al. (Konner et al., 2010)	2010	I	25 (at least one infusion of farletuzumab)	Escalating dose of 12.5–400 mg/m2 on days 1, 6, 15 and 22 of a 5-day cycle	Stable disease by Response Evaluation Criteria in Solid Tumors observed in 9 (36%) patients and CA-125 reduction in 4
Armstrong et al. (Armstrong et al., 2013)	2013	Phase II	47, (combination therapy with farletuzumab)	100 mg/m2, once weekly	Total or partial ORR was 75% with combination therapy
Vergote et al. (Vergote et al., 2016)	2016	III	1,091, (placebo group n = 352, farletuzumab 1.25 mg/kg group n = 376, farletuzumab 2.5 mg/kg group n = 363)	1.25 mg/kg or 2.5 mg/kg	Placebo group, farletuzumab 1.25 mg/kg group, farletuzumab 2.5 mg/kg group: PFS 9.0, 9.5, 9.7 months; OS 29.1, 28.7, 32.1 months, respectively
Herzog et al. (Herzog et al., 2023)	2023	II	214, (farletuzumab plus chemotherapy group n = 142, placebo plus chemotherapy group n = 72)	5 mg/kg weekly	Farletuzumab plus chemotherapy group, placebo plus chemotherapy: PFS 11.7, 10.8 months, respectively

ORR, overall response rate; PFS, progression-free survival; OS, overall survival.

### 5.3 Vintafolide

Vintafolide (MK-8109; EC145) is a water-soluble folate that is conjugated with deacetylvinyl-blastine monohydrase (DAVLBH). DAVLBH destabilizes microtubules, thereby disrupting mitotic division and leading to cell death. Vintafolide is a folate receptor ligand and has potent activity against xenograft tumors expressing FR (Parker et al., 2005; Reddy et al., 2007). Despite the potential use of vintafolide also in ovarian cancer, there are still very few studies in the literature determining its effectiveness.

In a 2012 phase I study, the goal was to determine the effectiveness and safety of EC145 in patients with refractory solid tumors. EC145 was administered as an intravenous bolus or 1-h infusion. The most common side effects were constipation, nausea, fatigue and vomiting. Of the 4 patients with ovarian cancer, 1 patient had one partial response to treatment (LoRusso et al., 2012). Evidence indicating the potential effectiveness of vintafolide in patients with ovarian cancer was the basis for further studies.

PRECEDENT is a randomized, phase II trial whose aim was to compare the effectiveness of vintafolide in combination with pegylated liposomal doxorubicin (PLD) compared to PLD administered alone. Furthermore, the study evaluated an imaging agent targeting FR that would have potential importance in selecting patients who would benefit most from this treatment. 162 patients with recurrent platinum-resistant ovarian cancer were enrolled and assigned in a 2:1 ratio to receive PLD with or without vintafolide 2.5 mg intravenously 3 times per week during weeks 1 and 3. The study results showed that the median PFS was 5.0 months for the group receiving vintafolide plus PLD and 2.7 months for the group receiving PLD alone. Interestingly, in this study, patients with FRα-positive tumors benefited from this combination therapy. Moreover, etharfoliatide has been shown to be helpful for imaging identification (Naumann et al., 2013). However, the phase 3 PRECEDENT trial was stopped due to failure to achieve the primary PFS result (Vergote et al., 2015). In 2016, the results of the phase II PRECEDENT trial were published, which showed that FR status does not matter regarding side effects in combination therapy with vintafolide + PLD or PLD alone in patients with platinum-resistant ovarian cancer (Herzog et al., 2016).

The clinical trials describing the efficacy of vintafolide in patients with ovarian cancer are presented in Table 10.

**TABLE 10:** Clinical trials with vintafolide in ovarian cancer.

Name of the study	Year of the study	Phase of the study	Research group	Dose of vintafolide	Results
Lorusso et al. (LoRusso et al., 2012)	2012	I	32	2.5 mg intravenously on days 1, 3, and 5 and days 15, 17, and 19 of each 28-day cycle	Acceptable safety profile; 1 (out of 4) patient with ovarian cancer had partial response to treatment
PRECEDENT (Naumann et al., 2013)	2013	II	162, (vintafolide plus doxorubicin group n = 109, doxorubicin alone group n = 53)	2.5 mg intravenously 3 times per week during weeks 1 and 3	Vintafolide plus doxorubicin group, doxorubicin alone group: PFS 5.0, 2.7 months, respectively

PFS, progression-free survival.

## 6 Immunotherapy

Strategies targeting the immune system in case of treating solid tumors, including ovarian cancers gave new hopes for patients, especially those who suffer from recurrences. Except for the evidence that ovarian cancers are immunogenic tumors, they belong to the group, for which immunotherapy did not show positive impact (Zhang et al., 2003; Kandalaft et al., 2012; Disis et al., 2019; Varga et al., 2019; Pujade-Lauraine et al., 2021). The reasons are still not known for sure, although it is speculated that responsible for it are potent immunosuppressive signals, which dominate the tumor microenvironment of ovarian tumors (Chen et al., 2022). Furthermore, it can be caused by the expression of many immune checkpoints, and the coexistence of a low tumor mutational burden with a dearth of neoantigens. (The Cancer Genome Atlas Research Network, 2011; PedBrain et al., 2013; Zamarin et al., 2020a). Nevertheless, there appear more and more new clinical trials that focus on testing different doses or combining standard methods with new ones, which may give better responses. So far researched immunotherapeutic approaches include, among others usage of checkpoint inhibitors, oncolytic viruses, reactive T cells and dendritic cells.

Selected results related to described therapies options are presented in Table 11, Table 12, Table 13. For these ones, which were not included in the table, results are presented in the text.

**TABLE 11:** Results of the selected studies examining checkpoint inhibitors in treating ovarian cancer.

Name of the study	Year of the study	Phase of the study	Research group	Dose	Results
NRG GY003 (Zamarin et al., 2020b)	2020	II	100, (treatment 1- nivolumab n = 49, treatment 2 - nivolumab plus ipilimumab n = 51)	Nivolumab 3 mg/kg iv. every 2 weeks or nivolumab 3 mg/kg iv. plus ipilimumab 1 mg/kg iv. every 3 weeks	Treatment 1, treatment 2: OS 21.8, 28.1 months; PFS 2.0, 3.9 months, respectively
Lee et al. (Lee et al., 2020)	2020	II	26 (all were given pembrolizumab)	Pembrolizumab 200 mg iv.) every 3 weeks and PLD 40 mg/m2 iv. every 4 weeks	Median PFS—8.1 (1.7–14.7) months and median OS was 18.3 (9.4–31.5) months
TPIV200 (Zamarin et al., 2020a)	2020	II	27	Durvalumab 750 mg intravenously on days 1 and 15 in cycles 1–12, and TPIV200 (500 µg per peptide; Marker, ref IB) admixed with GM-CSF (125 μg; Sargramostim) via three intradermal injections in the upper extremities on day 1 in cycles 1–6	The median PFS was 2.8 months (2.5–∞), OS was 21 months (13.5–∞)
JAVELIN Ovarian 200 (Pujade-Lauraine et al., 2021)	2021	III	566, (avelumab plus PLD n = 188, PLD n = 190, avelumab n = 188)	Avelumab (10 mg/kg iv. every 2 weeks), avelumab plus PLD (40 mg/m2 iv. every 4 weeks), or PLD	Median PFS (3.7 months combination group, 3.5 months in PLD group and 1.9 months in the avelumab group), overall survival (18.4 months vs. 18.2 months vs. 17.4 months)
JAVELIN Ovarian 100 (Monk et al., 2021)	2021	III	998, (avelumab n = 332, avelumab combination n = 331, and control n = 335)	Chemotherapy (carboplatin plus paclitaxel) followed by avelumab (10 mg/kg iv. every 2 weeks; avelumab maintenance group); chemotherapy plus avelumab (10 mg/kg iv. every 3 weeks) followed by avelumab maintenance (avelumab combination group); or chemotherapy followed by observation (control group)	Median PFS (16.8 months with avelumab maintenance, 18.1 months with avelumab combination treatment, and 18.2 months with control treatment)
NCI-2015–01910 (Konstantinopoulos et al., 2020)	2021	II	70	Gemcitabine iv. (1,000 mg/m2 during 30 min) on day 1 and day 8 of each 21-day cycle, either alone or in combination with intravenous berzosertib (210 mg/m2 during 1 h) on day 2 and day 9 of each 21-day cycle	Median PFS was 22,9 weeks (17.9–72.0) for gemcitabine plus berzosertib and 14.7 weeks for gemcitabine alone
CCR4420 (Papadatos-Pastos et al., 2022)	2022	I	34	Guadecitabine (45 mg/m2 or 30 mg/m2, administered subcutaneously on days 1–4), with pembrolizumab (200 mg administered iv. starting from cycle 2 onwards) every 3 weeks	PFS achieved for ≥24 weeks
CLEE011XUS28T (Coffman et al., 2022)	2022	I	35	Ribociclib, dosing levels groups: (a) 200 mg, (b) 400 mg, (c) 600 mg	Median PFS - 11.4 months
KGOG3046 (Park et al., 2023)	2023	II	23	Three cycles of durvalumab (1,500 mg) and tremelimumab (75 mg) with NAC, followed by IDS; after surgery, three cycles of durvalumab (1,120 mg) and adjuvant chemotherapy followed by durvalumab maintenance (1,120 mg [total 12 cycles]) were administered	The median PFS was 17.5 months, and the median OS was not reached in the modified ITT population

iv. - intravenous; PFS, progression-free survival; OS, overall survival; PLD, pegylated liposomal doxorubicin; GM-CSF, granulocyte-macrophage colony-stimulating factor; NAC, neoadjuvant chemotherapy; IDS, interval debulking surgery; ITT, intent to treat.

**TABLE 12:** Results of the selected studies examining oncolytic viruses in treating ovarian cancer.

Name of the study	Year of the study	Phase of the study	Research group	Dose	Results
Galanis et al. (Galanis et al., 2010)	2021	I	21	MV-CEA virus every 4 weeks for up to 6 cycles at seven different dose levels (103–109 TCID50)	Median survival was 12.15 (1.3–38.4 months), best objective response was dose-dependent disease stabilization was observed in 14 of 21 patients and with median duration of 92.5 days (54–277 days)
Cohn et al. (Cohn et al., 2017)	2017	II	108	Paclitaxel (80 mg/m2 intravenously days 1, 8, and 15 every 4 weeks) or the combination of paclitaxel (80 mg/m2 intravenously days 1, 8, and 15) plus reovirus 3 × 1010 TCID50/day intravenously on days 1–5, both every 4 weeks until disease progression or toxicity	Median PFS was 4.3 months for paclitaxel and 4.4 months for paclitaxel plus reovirus
ColoAd1-2001 (Moreno et al., 2021)	2021	I	38	Enadenotucirev iv. (1 × 1,012 viral particles; days 1, 3 and 5 every 28-day for two cycles) plus paclitaxel (80 mg/m2; days 9, 16 and 23 of each cycle)	4-month PFS rate for 20 patients who received intravenous enadenotucirev plus paclitaxel was 64% (median 6.2 months) and 63% of the patients experienced treatment-emergent adverse event - first of all neutropenia (21%)

MV-CEA, carcinoembryonic antigen-expressing oncolytic measles virus derivative; TCID, tissue culture infectious dose; PFS, progression-free survival.

**TABLE 13:** Results of the selected studies examining T cell immunotherapy in treating ovarian cancer.

Name of the study	Year of the study	Phase of the study	Research group	Dose	Results
Kershaw et al. (Kershaw et al., 2006)	2006	I	14	3 × 109–5 × 1,010 transduced T cells	There were observed some grade 3 and 4 toxicities in the group with high-dose usage of IL-2; in the group, which received T cells without IL-2, patients experienced relatively mild side effects; there was not observed any tumor burden in patients
Dobrzanski et al. (Dobrzanski et al., 2012)	2012	Phase I/II	7	108–109 T cells per infusion (i.e. 1–4 × 108 cells/m2)	There was observed enhanced patient survival in 3 monthly treatment cycles (3->84 months)

IL, interleukin.

In the case of checkpoint inhibitors, they work by blocking the inhibitor receptors on the surface of T cells, or their corresponding ligands. Moreover, they prevent exhaustion and promote activation of T cells to enhance tumor detection and destruction (Zamarin et al., 2020a). Although they achieve high effectiveness in the treatment of malignancies, like melanoma or renal clear cell carcinoma (Zamarin et al., 2020b), as for treating ovarian cancers their effectiveness alone induces clinical responses in <10% (Ferrara et al., 2004; Xu et al., 2012). Much more enhanced antitumor activity was demonstrated when testing the simultaneous use of a combination of checkpoint inhibitors targeting PD-1 and CTLA-4, than using them alone (Curran et al., 2010; Duraiswamy et al., 2013; Selby et al., 2016). What is more it was observed that chemotherapy increases tumor responsiveness to checkpoint inhibitors, in the AURELIA trial (research group:361; bevacizumab plus chemotherapy vs. chemotherapy alone: ORR, 30.9 *versus* 12.6% [*p* < 0.001] and median PFS, 6.7 vs. 3.4 months [*p* < 0.001]) (Pfirschke et al., 2016; Pujade-Lauraine et al., 2021). Among them, doxorubicin turned out to be an inducer, which triggers an adaptive immune response (Zitvogel et al., 2013).

Another hope gives usage of viral vectors and dendritic cells. First of them act by selective replication in cancer cells, which leads to local amplification and ultimately to cell death (Chen et al., 2022). In the study by Moreno V. et al. it was showed that usage of tumor selective adenovirus enadenotucirev increased tumor immune-cell infiltration in platinum-resistant ovarian cancer (Moreno et al., 2021). As for autologous dendritic cells, they can be expanded, activated, and loaded with a source of tumor-associated antigens (TAAs) *ex vivo* (Cibula et al., 2021). Loading them with many different of TAAs affects both the reduction of the risk of probability of immune evasion via antigen loss, as well as increasing the potency of immunization. Moreover, it plays a role in generating a polyclonal T-cell response against malignant cells (Keskin et al., 2019). In the II phase study SOV02 it turned out that DC combined with chemotherapy influenced significantly OS prolongation (13,4 months) and enhanced surrogate antigen-specific T-cell activity, but did not improve PFS (Cibula et al., 2021). Research group included 71 patients (39 received 1 mL aliquot of DCVAC/OvC) (Selby et al., 2016).

Research has also been conducted on the use of adoptive T cell immunotherapy. It is based on the use of naturally existing tumor-reactive T cells already present within the tumor, collecting them from the patient, then their activation and expansion *in vitro*, and after that reintroducing them into the patient’s body (Andersen et al., 2015). This treatment has extensive clinical experience in patients with metastatic melanoma. Nevertheless, the study by Dobrzański M.J. et al. showed that the best effects were achieved, when treatment was combined with conventional modalities and burden was minimal (Dobrzanski et al., 2012).

Another promising direction is also epigenetics, which, through the use of hypomethylating agents like decitabine and 5-azacitadine (Chen et al., 2022), opens the possibility of increasing the immunogenicity of ovarian cancer and augmenting the activity of immune checkpoint inhibitors (Kershaw et al., 2006; Dobrzanski et al., 2012).

One of the other new possible directions is also usage of the trastuzumab—monoclonal antibody. It has been approved so far for treatment of HER2-expressing breast cancer and HER2-positive gastric or gastroesophageal junction adenocarcinoma in the United States and European Union, and for HER2-mutant non-small cell lung cancer in United States and Japan (Omar et al., 2015). In the second phase open-label DESTINY-PanTumor02 trial (evaluation of the Efficacy and Safety of Trastuzumab Deruxtecan for the Treatment of Selected HER2 Expressing Tumors) in patients with ovarian tumor (n = 40) OS was 13.2 months in the whole group and 20.0 months in the group with HER2 IHC 3+ expression and objective response rate (ORR) was 45% (Meric-Bernstam et al., 2024). These meaningful survival outcomes, which were also demonstrated in endometrial and cervical cohorts, can play role in revolutionizing the treatment of HER2-expressing solid tumors.

What is more, in the study of Yang Y. et al. (Yang et al., 2018) efficacy of trastuzumab alone was evaluated in comparison to combined medication of abraxane (paclitaxel) and trastuzumab amid a group of the 80 patients with recurrent ovarian cancer. Results showed that combination of the two medications vs usage of trastuzumab alone had higher OS (7.3 vs 7 months, *p* = 0,63) and lower incidence of neutropenia (40,5% vs 51,2%).

Most of the described studies are in the early phases. Nevertheless, results showed that, new ways of approaching microenvironment of ovarian tumors, can be used successfully in coping with its immunosuppressive signals. These directions, especially—epigenetics can be the future of the treatment of the most aggressive ovarian tumors, including recurrences.

## 7 Discussion

Despite numerous treatment methods available, ovarian cancer is still associated with the risk of recurrence and metastasis. These data raise questions: what changes in the treatment should be made and what should future studies focus on to increase the effectiveness of ovarian cancer treatment?

Bevacizumab remains an important method in the treatment of ovarian cancer. However, it turns out that not only angiogenesis, but also lymphangiogenesis is an important process in the development of cancer. Bevacizumab affects blood vessels, but not lymphatic vessels. Moreover, it turns out that, to our knowledge, there are no studies that would examine the impact of lymph node metastases on the course of ovarian cancer treatment with bevacizumab. Therefore, despite many promising studies using bevacizumab in ovarian cancer, there is a great need to investigate its effect on ovarian cancer (Sopo et al., 2020).

The results of studies confirm the validity of using PARP inhibitors in the treatment of ovarian cancer. Based on the results, PARP inhibitors appear to provide the most favorable efficacy in patients with BRCA1/BRCA2 mutations who test positive for HRD. The results of the SOLO1 study indicate an improvement in OS in patients with a BRCA mutation after receiving olaparib, and the results of the PAOLA-1 study indicate an improvement in OS in HRD-positive patients (DiSilvestro et al., 2023; Ray-Coquard et al., 2023). Therefore, the standard in the diagnosis of patients should be the study of biomarkers, which will allow us to determine the group that will benefit the most from this therapy. There are the AstraZeneca AZ HRR tests for homologous repair mutations, the Myriad MyChoice test for single nucleotide polymorphisms, which can be used to determine whether a patient is HRD-positive or negative (AlHilli et al., 2016).

In terms of side effects, taking into account the results of available studies, the safety profile of PARP inhibitors appears to be similar. In the ARIEL3, ATHENA and ARIEL4 studies, the most common side effects associated with the use of rucaparib were anemia or decreased hemoglobin (Clamp et al., 2021; Kristeleit et al., 2022; Monk et al., 2022). In turn, the low number of adverse events associated with the use of niraparib was due to the fact that a large proportion of patients were initially treated with niraparib at a lower dose of 200 mg daily. The NORA study is, to our knowledge, the first such study to establish an individual dosing regimen for this drug (Wu et al., 2021). Therefore, individualization of dosage is important to reduce the number of possible side effects and thus improve the quality of life of patients. It seems that the risk of MDS/AML with the use of PARP inhibitors is rather low in newly diagnosed patients. This risk is higher when treating recurrent ovarian cancer. It is therefore important in this case to monitor patients for this side effect to determine exactly which group is actually at risk of MDS/AML.

Currently, research is ongoing on the effectiveness of using PARP inhibitors in combination with other treatment methods. Although the use of PARP inhibitors together with chemotherapy may cause increased toxicity and side effects, there are combinations such as PARP inhibitors with antiangiogenic therapy or immunotherapy that may be optimal in the future.

Taking into account the fact that both angiogenesis inhibitors and PARP inhibitors do not significantly prolong OS in patients with ovarian cancer, it is necessary to conduct further research on new diagnostic and therapeutic strategies for ovarian cancer. The expression of FRα on the surface of ovarian cancer cells is an important premise for conducting research on the effectiveness of folate receptor alpha inhibitors.

The study results indicate a significant benefit from treatment with folate receptor alpha inhibitors in patients whose tumors showed positive FRα expression. In the phase II PRECEDENT trial, the greatest benefit from vintafolide was achieved by patients with 100% positive FRα expression (Naumann et al., 2013). The same is also confirmed by the results of the randomized phase III FORWARD trial comparing chemotherapy with MIRV with IC chemotherapy (Moore et al., 2021).

Mirvetuximab soravtansine seems to have an extremely beneficial effect, and its effect may be greater than that of farletuzumab or vintafolide. MIRV has both an ADC molecule and a cytotoxic agent, which provides both good pharmacokinetic properties and tumor cell death. Moreover, it has an extended half-life, which affects the delivery of the cargo to the site where the tumor is (Kovtun et al., 2006).

Both mirvetuximab, farletuzumab and vintafolid have a good safety and tolerability profile. The most common side effects can be quickly recognized and managed. Such results may constitute a reason to conduct further studies on the combination of folate receptor alpha inhibitors with other treatment methods, e.g., bevacizumab or pembrolizumab. In fact, the FORWARD II study indicates the effectiveness of the combination of MIRV with bevacizumab or pembrolizumab (O Malley et al., 2020). Moreover, it should be noted that folate receptor alpha inhibitors have the ability to disrupt microtubules, and taxanes also have a similar mechanism of action. Perhaps, further research will allow in the future to replace treatment with taxanes in patients with FRα-positive tumors.

Interestingly, it turns out that the use of PARP inhibitors is likely not limited only to patients with mutations in DNA repair pathways, but also to patients with newly diagnosed advanced ovarian cancer. Giannini et al. in their review, they critically assessed the PRIMA, PRIME and ATHENA-mono studies regarding the use of PARP inhibitors in newly diagnosed ovarian cancer (Giannini et al., 2023).

So far, the results of studies using immuno-oncology approaches have brought limited success in the case of treating ovarian cancer. Hopes for enhancing their activity lies in their combination (for example, two checkpoint inhibitors), or in combination with conventional methods, like chemotherapy. Another hopes is increasing immunogenicity of the tumor before using these methods, by epigenetic approaches like the use of hypomethylating agents.

Although our narrative review flexibly reports the latest treatment outcomes for ovarian cancer patients, it has limitations. First, despite the methodology used, the article may lack systematic checking for bias. Secondly, we conducted a review of work articles with no time restriction. Moreover, our review is selective, which may make it difficult to critically evaluate the articles included in our manuscript.

Our review focused on existing and new research related to the treatment of ovarian cancer. The choice of appropriate treatment also involves knowledge of numerous biomarkers responsible for the development and course of the disease. Those responsible for this include, among others: signal transduction pathways, growth factor receptors, angiogenic processes, cell cycle regulators and drug delivery systems. Further research on the molecular changes occurring in ovarian tumors is definitely necessary to develop new therapeutic strategies or improve existing ones.

In summary, the ovarian cancer environment is extremely complicated due to tumor heterogeneity, different histological and molecular types and mutations. For this purpose, a detailed analysis of biomarkers and targeted therapies is extremely important. Future research should aim to investigate biomarker analysis methods in patients with ovarian cancer, which will allow for the selection of the treatment method from which a given patient will benefit the most. Personalized and individualized treatment should be the primary goal of clinicians. While bevacizumab is still an important treatment method in ovarian cancer, its effect on lymph node metastases is still questionable. To determine which group of women is most at risk for side effects associated with PARP inhibitors such as MDS/AML, it is important to monitor patients during and after treatment. When it comes to immunotherapy, hopes are associated with the combination of, for example, two checkpoint inhibitors or their combination with other methods such as chemotherapy. Although none of the current studies have shown that a given treatment method will cure ovarian cancer, great hopes are still associated with new clinical trials on combination therapies, studies of biomarkers or the tumor microenvironment and immunosuppressive pathways.

The standard treatment of the primary ovarian cancer is surgical removal of the tumor and assessment of the cancer’s advancement along with possible adjuvant chemotherapy. Nevertheless there are therapies and treatment strategies, which give new hopes for patients, including: antiangiogenic therapy, PARP inhibitors, folate receptor alpha inhibitors, or immunotherapy (checkpoint inhibitors, adoptive T cells, oncolytic viruses) or epigenetics methods like using hypomethylating agents.

Bevacizumab has the ability to bind to all isoforms of vascular endothelial growth factor A (VEGF-A). In this way, activation of the VEGF signaling pathway is blocked, limiting the formation of new vessels in the tumor, which prevents further tumor growth. At the same time, bevacizumab reduces tumor vascular permeability and interstitial fluid pressure (IFP), resulting in greater drug convection within the tumor. Both mechanisms of action of bevacizumab limit local tumor progression and metastasis.
